# Physical activity moderates the relationship between hot flashes and arterial wave reflection in perimenopausal females

**DOI:** 10.14814/phy2.70918

**Published:** 2026-05-21

**Authors:** Tint Tha Ra Wun, Jo Sophia Brunzelle, Lorna Murphy, Randi L. Garcia, Lynnette Leidy Sievert, Sarah Witkowski

**Affiliations:** ^1^ Department of Exercise and Sport Studies Smith College Northampton Massachusetts USA; ^2^ Department of Psychology and Program in Statistical and Data Sciences Smith College Northampton Massachusetts USA; ^3^ Department of Anthropology University of Massachusetts Amherst Massachusetts USA

**Keywords:** arterial wave reflection, hot flashes, perimenopause, physical activity

## Abstract

Subclinical CVD risk factors are associated with hot flashes (HF). Habitual physical activity (PA) reduces CVD risk in part due to effects on blood vessel characteristics. The objective was to evaluate associations between arterial wave reflection (WR), HFs, and PA in perimenopausal people and determine whether PA moderates the relationship. Objective HF were related to higher WR (*r* (51) = 0.31, *p* = 0.02). Moderate to vigorous PA was negatively associated with the augmentation index normalized for a heart rate of 75 beats/min (AIx75) (*r* (57) = − 0.42, *p* = 0.0009) and WR (*r* (59) = − 0.34, *p* = 0.008). Inactivity time was correlated with WR (*r* (59) = 0.35, *p* = 0.005). Simple slope analysis showed that moderate‐intensity PA (MPA) affected the relationship between HF and WR (HFxMPA, *ꞵ*[SE] = −0.37[0.17], *p* = 0.04), as the association was significant at lower levels of moderate PA (‐1SD from mean MPA, *ꞵ*[SE] = 23.33[7.52], *p* = 0.003) but not higher levels (+1SD from mean MPA, *ꞵ*[SE] = 2.48[6.1], *p* = 0.69). Findings suggest that moderate to vigorous PA is an important strategy to reduce CVD risk related to HF in perimenopause.

## 1 INTRODUCTION

Perimenopause is a transitional time around the final menstrual period characterized by variable menstrual cycle length, irregularities in reproductive hormones, and symptoms such as hot flashes (HF), night sweats, sleep disturbances, mood changes, genitourinary, and sexual function changes. A growing body of literature suggests that the risk for cardiovascular disease (CVD) increases more dramatically during the menopause transition than at other times during the lifespan (El Khoudary et al., 2020; El Khoudary & Nasr, 2022). The incidence of CVD in females exhibits a notable increase around perimenopause and the diagnosis of clinical CVD rises significantly after the final menstrual period (Ryczkowska et al., 2022; Kamińska et al., 2023). Thus, there is a need to understand and mitigate early changes in subclinical risk factors in peri‐menopausal females.

Hot flashes are a hallmark symptom of menopause, correlated with poorer quality of life, mental health, and functioning during the menopause transition (Williams et al., 2008). Hot flashes are reported by 70% of people who undergo menopause, a third of whom experience frequent or severe HF (Gold et al., 2006; Thurston et al., 2017; Williams et al., 2008) and are a primary reason females seek medical advice during menopause (Hess et al., 2012; Padilla et al., 2018). Studies have linked the causation of HF to estrogen withdrawal and higher activity of kisspeptin/neurokinin B/dynorphin neurons in the preoptic area of the hypothalamus (Mittelman‐Smith et al., 2015; Rance et al., 2013). The frequency and severity of HF are associated with subclinical CVD risk factors, such as greater endothelial dysfunction (Thurston et al., 2017; Witkowski et al., 2025), greater intima‐media thickness (Thurston et al., 2011), and lower carotid artery compliance (Hildreth et al., 2018) although uncertainty remains as not all studies show clear associations (Lambrinoudaki et al., 2012; Yang et al., 2017). The majority of studies have assessed HF via self‐report which can be influenced by recall bias and awareness. Objective symptom measures can strengthen understanding of relations between HF and CVD risk.

Changes in arterial properties precede the clinical onset of CVD, and the cardiovascular consequences appear to be greater in females than males (Coutinho et al., 2013; Regnault et al., 2012). Wave reflection increases with aging to a greater degree in females than males. The menopause transition is independently associated with increased arterial stiffness (Khan et al., 2018; McEniery et al., 2005; Namasivayam et al., 2009; Russo et al., 2012; Samargandy et al., 2020; Vallée, 2025). Arterial pressure waveform analysis may complement and extend understanding of the nature of the development of CVD risk during the menopause transition as the consequences to increased arterial wave reflection may be worse in females. Increased pulsatility and wave reflection result in greater potential for end organ damage and left ventricular hypertrophy via increased afterload (Weber et al., 2012). Wave augmentation and its forward and reverse wave components have been associated with various CVD outcomes (Chirinos et al., 2012; Cooper et al., 2015; Desbiens et al., 2022; Norton et al., 2024). The Strong Heart Study reported that central pulse pressure ≥50 mmHg indicates adverse CVD outcome in both males and females. (Roman et al., 2009) Some data suggest that females may be more vulnerable to the effects of aortic stiffness on the heart (Coutinho et al., 2013; Shim et al., 2011). Costa‐Hong et al. showed that indexes of aortic wave reflection (WR) in hypertensive menopausal females were worse compared to hypertensive same‐age males and younger females (Costa‐Hong et al., 2018). Improved understanding of arterial pressure waveforms around the transition to menopause may extend knowledge of factors that contribute to the increasing CVD risk in midlife females.

One factor that is often neglected in CVD studies for the perimenopausal population is habitual physical activity (PA). PA has been proven effective in the prevention and treatment of CVD in many populations (Haskell et al., 2007); engagement in regular PA and reduction in sedentary behaviors are widely recommended (Coll‐Risco et al., 2018; Lin et al., 2020). The World Health Organization 2020 guideline on PA and sedentary behavior recommends weekly activity of at least 150–300 min of moderate‐intensity aerobic activity, 75–150 min of vigorous‐intensity aerobic activity, or some equivalent combination of moderate‐ and vigorous‐intensity aerobic activity and limiting sedentary time (Bull et al., 2020). In line with the U.S. Department of Health and Human Services (HHS) Physical Activity Guidelines for Americans (Piercy et al., 2018), the International Menopause Society recommends at least 150 min of moderate‐intensity aerobic activity and two or more days of strength/resistance training every week (Baber & Panay, 2016). Previously, increased central arterial stiffness was correlated with physical inactivity in healthy females with no age‐related increase of arterial blood pressure (BP) (Tanaka et al., 1998). Importantly, the age‐associated increase of carotid augmentation index (AIx) and aortic pulse wave velocity (PWV) was absent in physically active females (Tanaka et al., 1998). More recently, a study by Lan et al. (Lan et al., 2023) showed that PWV increased with age but was inversely related to exercise intensity, demonstrating potential for PA to attenuate arterial changes around menopause. Given rising CVD risk around the menopause transition, assessing the influence of physical activity on arterial pressure waveforms in perimenopause requires further evaluation.

The goal of this study was to use objectively assessed outcomes to determine the relationship between arterial waveform analysis parameters, hot flashes, and physical activity and to investigate whether physical activity moderates the association between arterial waveform properties and hot flashes in healthy perimenopausal people. We hypothesized that more objectively measured hot flashes would be associated with arterial waveform parameters, specifically higher Augmentation Index normalized to a heart rate of 75 (AIx75), WR, and PWV. In regression models, we hypothesized that hot flashes and physical activity would be significant predictors of arterial waveform parameters and PWV. Considering physical activity, we hypothesized that greater physical activity would mitigate the negative relation between hot flashes and arterial waveform parameters and PWV.

## 2 MATERIALS AND METHODS

All protocols were approved by the Smith College Institutional Review Board (IRB ID: 18‐108) and participants provided a written informed consent prior to any study procedures. The study was in accord with the Declaration of Helsinki.

### 2.1 Screening

Healthy females aged 43–54 years were recruited for our study. All participants were perimenopausal as defined by the Stages of Reproductive Aging Workshop (STRAW+10) guidelines. Under these guidelines, perimenopause is defined as changes in menstrual cycle length of ≥7 days than normal up to less than 1 year of amenorrhea. (Harlow et al., 2012) The recruitment took place in Western Massachusetts via public events, social media, and local flyering. Participants had to report stable physical activity levels for at least the past 2 years to be included. The exclusion criteria included a history of CVD, chronic menstrual irregularities (e.g., polycystic ovary syndrome), surgical menopause, use of intrauterine devices, prescription of menopausal symptom treatment, oral contraceptives in the past 6 months, hormone therapy, currently pregnant or lactating, or currently smoking or had smoked in the last 6 months.

Participants with elevated CVD risk were excluded. A fasting blood sample was drawn to measure total cholesterol (TC), triglycerides (TRG), low‐density lipoprotein cholesterol (LDL‐C), high‐density lipoprotein cholesterol (HDL‐C), and fasting plasma glucose. Participants were ineligible if fasting plasma lipids were at any of the following levels: LDL‐C > 160 mg/dL, HDL‐C < 40 mg/dL, TRG >200 mg/dL, TC >240 mg/dL, or fasting plasma glucose >126 mg/dL. Additionally, height, weight, and resting BP were measured. Body mass index (BMI) was computed from height and weight (kg/m^2^). Participants were excluded from the study if resting BP was >140/>90 mmHg. If BMI was <18.5 or >35 kg/m^2^, participants were evaluated for other CVD criteria and were eligible to participate if they were within limits for LDL‐C < 160 mg/dL, HDL‐C > 40 mg/dL, TRG <200 mg/dL, TC <240 mg/dL, and fasting plasma glucose <126 mg/dL.

Of 208 people screened, 77 eligible people enrolled in the study. Data from participants with incomplete data (e.g., missing HF data), changes in menstrual cycle or medication, or those who voluntarily withdrew were not included in the analysis. Ultimately, data from 61 participants were used in this analysis.

### 2.2 Arterial wave and velocity analysis

Arterial wave and velocity variables were measured according to current published recommendations (Sharman et al., 2017; Townsend, 2017) using a non‐invasive tool, the SphygmoCor® XCEL system (AtCor Medical, Naperville, IL, USA) along with the SphygmoCor® software suite (AtCor Medical, Naperville, IL, USA). The SphygmoCor® XCEL system can derive central aortic pressure waveform from the pressure pulse recorded non‐invasively at a peripheral site (Butlin & Qasem, 2017). A familiarization trial was performed for all participants during the screening visit. On the testing day, all assessments took place in the morning to control diurnal variation. For participants who experienced menstrual periods, arterial assessment was performed on Days 2–5 of the menstrual cycle. Before the arterial assessment visit, participants were instructed to fast for at least 6 h, to refrain from alcohol, caffeine, exercise, and smoking for 12 h, to avoid any pain medication and phosphodiesterase inhibitors for 24 h, and to prevent taking any vitamins and supplements 72 h prior to data collection.

Participants were placed in a supine position and rested for at least 10 min in a temperature‐controlled room before the assessment. All measurements were made on the right side using the SphygmoCor® XCEL. Trials recorded systolic BP (SBP) and diastolic BP (DBP) by measuring a volumetric displacement with an inflation cuff at the brachial artery. BP values were used to obtain a brachial waveform, which was analyzed by the SphygmoCor® software to produce a central aortic waveform. The validity of the measurement was determined by quality control parameters built into the software and the assessment was repeated if quality control was <95%.

Arterial and central waveform variables for this analysis included forward wave (*P*_f_), backward wave (*P*_b_), wave reflection (WR), augmentation index (AIx), and AIx normalized for a heart rate of 75 beats/min (AIx75). WR was calculated as the difference between *P*_f_ and *P*_b_, expressed as a percentage of the total *P*_f_ ([*P*_f_–*P*_b_/*P*_f_] * 100%). AIx was the degree to which the pressure wave peak was above the peak incident pressure wave due to reflected pressure waves, calculated as the difference between the first and second peaks of the central arterial waveform as a percentage of the pulse pressure (SBP ‐ DBP). AIx75 was the AIx value corrected for heart rate at 75 beats/min. While both calculate central arterial waveform analysis, WR is not dependent on heart rate or timing of the inflection point like AIx75, but is dependent on the geometry and stiffness of the arterial tree distal to the aortic arch (Butlin & Qasem, 2017).

Carotid‐femoral PWV was captured with a tonometer placement at the carotid artery and an inflation cuff at the femoral artery. The point with the strongest carotid pulse was used for the tonometer placement. Distances between the carotid pulse point to the sternal notch and between the sternal notch and top edge of the femoral cuff were directly measured and entered into the software. Trials recorded BP waveforms at the respective sites and the SphygmoCor® software was used to impute PWV. PWV is calculated as the ratio of the distance between the carotid and femoral arterial sites and the time delay of the pulse between these sites. Quality control by the SphygmoCor® software checks for the consistent waveforms for both carotid and cuff tracing and the assessment was repeated if quality control was poor.

### 2.3 Hot flash assessment

Objective measurement of HF can overcome issues of subjective reporting such as recall bias and awareness (Thurston et al., 2017). HF were measured via sternal skin conductance using an ambulatory device (Biolog monitor, UFI, Morro Bay, CA, USA) (Sievert, 2013). Silver/silver chloride electrodes placed 4 inches apart across the mid‐sternum used a 0.5 constant voltage circuit to measure skin conductance. In addition to objective HF, subjective HF were recorded over the 24 h. Participants were instructed to push a button on the device when they felt a HF to mark a subjective HF. Furthermore, a paper log was provided to report time‐in‐bed and time‐out‐of‐bed and subjective HF retrospectively if pressing the button was not possible at any point they felt a HF.

The monitor recordings were analyzed using the 3991× GPP Biolog DPS software (version 1.2) and FlashTrax software (version 2.1) (UFI, Morro Bay, CA, USA). The criteria for objective HF were as follows: an increase in conductance of 2 μ℧ over 30 s with a 20‐min post‐HF lockout period. In addition to objective HF that met the criteria, the recordings were also reviewed manually to record objective HF which did not meet established criteria but had a characteristic change in sweating pattern and was accompanied by the participant's subjective report (Witkowski et al., 2025; Witkowski, White, Shreyer, Brown, & Sievert, 2024; Witkowski, White, Shreyer, Garcia, et al., 2024). Only valid records with at least 9 h of wear time were included in data analysis.The HF variable assessed in this analysis is total objective HF rate. Total objective HF count was the number of objective HFs experienced during the total wear time. Total objective HF rate was calculated as the number of all objective HF counts divided by the total monitor wear time. All participants were included in the analysis regardless of whether they experienced HF symptoms during the 24‐h monitoring period.

### 2.4 Physical activity

Participants completed the International Physical Activity Questionnaire (IPAQ) short form to assess subjective PA. Objective PA was measured using the GT3X ActiGraph accelerometer (wGT3XP‐BT: ActiGraph, Pensacola, FL, USA) worn on the non‐dominant wrist of the participant for seven consecutive days except for removal during water‐related activities (e.g., swimming, showering). A valid wear day was considered as 21.6 h to provide sufficient reliability (>90%) (Riddoch et al., 2007) regardless of the day of the week (i.e., weekday vs. weekend). Monitors were programmed to measure 1‐min epochs, and the raw acceleration signal output was downloaded using the ActiLife software (version 6.8.1; ActiGraph, Pensacola, FL, USA). The recordings were analyzed using the GGIR R package (version 2.9‐0) (Migueles et al., 2019; van Hees et al., 2013, 2015) to calculate average minutes of PA at five different PA categories per day. Activity categories calculated include sedentary (physical inactivity), light activity (LPA), moderate activity (MPA), and vigorous activity (VPA). Moderate‐vigorous (MVPA) was the sum of MPA and VPA minutes. Using the published cut‐point recommendations of Hildebrand et al. for adults aged 21–61 (Hildebrand et al., 2014, 2017), the cut‐point threshold for light activity was set as 44.8 (in milligravity, mg), moderate activity as 100.6 mg and vigorous activity as 428.8 mg. While PA was recorded for 7 days, the Heuristic algorithm looking at Distribution of Change in Z Angle (HDCZA algorithm) from the GGIR package was used to derive sleep time and only waking‐hour PA was considered in this study.

### 2.5 Blood hormone analysis

Serum blood samples were collected and used to assess 17β‐estradiol (E2, AbCam, Waltham, MA USA, Catalogue #ab108667) and follicle‐stimulating hormone (FSH, RayBiotech, Peachtree Corners, GA, USA, Catalogue #ELH‐FSH‐1) via ELISA as previously described (Witkowski et al., 2025). The blood collection was completed on the same day as the arterial assessment and followed the same restrictions described above. The 4‐parameter logistics standard curves were fitted for both measures (*R* ^2^ = 0.99) and the concentrations for the samples were calculated based on the curve parameters. If the CV% for the triplicates was higher than 10%, the replicate that was ±2 standard deviations from the mean of the other two replicates was excluded from average concentration calculation. The average coefficient of variation for samples was 4.9% ± 3.2 for E2 and 4.67% ± 2.60 for FSH. For E2, the minimum level of detection was imputed for samples with a concentration lower than the minimum detection threshold. For FSH, the conversion to International Unit (mIU/mL) from (pg/mL) was calculated using 1 mIU/mL = 70 pg/mL, per the manufacturer's recommendation.

### 2.6 Data analysis

All statistical analyses were performed using R (version 4.2.3, R Foundation for Statistical Computing, Vienna, Austria) and RStudio software (2023.06.2 + 561, Posit Software, PBC, Boston, MA).

All data and models were evaluated for adherence to assumptions of each statistical test. Averages and standard deviations were calculated for each variable (Table 1). Pearson correlations were used to measure associations between the dependent arterial waveform variables (i.e., WR and AIx75) with objective HF rate and different objective PA engagement variables (i.e., inactivity, LPA, MPA, VPA, and MVPA). Due to the skewed nature of HF data (i.e., non‐normal distribution), correlations were also evaluated using nonparametric Spearman's rank correlation.

**TABLE 1:** Participant characteristics.

Characteristic	Mean ± SD
Age (year)	49 ± 3
Height (cm)	164 ± 6
Weight (lb)	147 ± 28
BMI (kg/m^2^)	24.7 ± 4.9
HDL (mg/dL)	69 ± 14^b^
LDL (mg/dL)	105 ± 26^e^
TRG (mg/dL)	79 ± 32^e^
TC (mg/dL)	184 ± 30^a^
FPG (mg/dL)	88 ± 9^a^
SBP (mmHg)	111 ± 12
DBP (mmHg)	70 ± 7
E2 (pg/mL)	35 ± 35^b^
FSH (mIU/mL)	8 ± 6^b^
Objective HF count (count/wear day)	5 ± 6^d^
Objective HF rate (count/wear hour)	0.21 ± 0.26^d^
HF monitor weartime (hour)	22 ± 6^b^
Subjective PA (MET‐min/wk)	3281 ± 2621
Objective Inactivity (mins/day)	718 ± 74
Objective LPA (mins/day)	160 ± 41
Objective MPA (mins/day)	77 ± 30
Objective VPA (mins/day)	9 ± 14
Objective MVPA (mins/day)	86 ± 37
*P*_f_	23.32 ± 4.46
*P*_b_	13.84 ± 3.45
WR (%)	59 ± 10
AIx (%)	27 ± 14
AIx75 (%)	20 ± 15^b^
PWV (m/s)	5.4 ± 1.1^c^

*Note*: *n* = 61, unless otherwise noted.

Abbreviations: AIx, augmentation index; BMI, Body Mass Index; DBP, diastolic blood pressure; E2, 17β‐estradiol; FPG, fasting plasma glucose; FSH, follicle‐stimulating hormone; HDL, high‐density lipoprotein cholesterol; HF, hot flash; LDL, low‐density lipoprotein cholesterol; LPA, light physical activity; MPA, moderate physical activity; MVPA, moderate‐vigorous physical activity; PA, physical activity; *P*_b_, backward wave amplitude; *P*_f_, forward wave amplitude; PWV, pulse wave velocity; SBP, systolic blood pressure; TC, total cholesterol; TRG, triglycerides; VPA, vigorous physical activity; WR, arterial wave reflection.

^a^ *n* = 60.

^b^ *n* = 59.

^c^ *n* = 56.

^d^ *n* = 53.

^e^ *n* = 42.

Simple and multiple linear regression analyses were performed to determine the relationships between arterial waveform analysis, hot flashes, and physical activity variables. Multiple regression models with interaction terms were used to address whether PA moderated the relationships between HF and WR. Additional predictors known to influence arterial stiffness (i.e., age, BMI, and BP) were controlled in models. When covariates had multiple measures (e.g., SBP and DBP), the one measure having the strongest Pearson's correlation with the corresponding arterial waveform variable was included to avoid collinearity issues. Models were reported in the following manner: multiple linear regression models with different PAs levels separately (including covariates), multiple linear regression models with objective HF rate (including covariates), and multiple linear regression with an interaction between objective HF rate and MPA (interaction model with covariates included). Regression assumption and residual diagnostics, including normality and constant variance tests, were performed for all models to ensure adherence.

## 3 RESULTS

### 3.1 Participant characteristics

All participant demographic characteristics are reported in Table 1. Participants were healthy perimenopausal females free of CVD risk factors and had high PA levels. The mean age of participants at entry was 49 ± 3 years. Most participants were non‐Hispanic and of European ancestry. The average recorded BP, BMI, cholesterol, and glucose levels of the participants were within normal range for a general healthy population (Chung et al., 2010). There were three participants with BMI >35; however, they were otherwise within healthy limits for BP, cholesterol, and glucose levels. Both E2 and FSH levels of the participants varied widely, which was expected for perimenopause.

Among 53 participants with valid objective HF recordings, 71% of participants achieved at least one objective HF over a 24‐h period while the remaining did not experience HF. The average objective HF rate for the group was 0.21 HF/wear hour. The average WR for our group was 59% ± 10, AIx was 27% ± 14, and AIx75 was 20% ± 15, and PWV was 5.4 m/s ± 1.1. The recorded average MVPA of the group was 86 min/day ±37 which was higher than the recommended PA levels for this age group (Baber & Panay, 2016; Bull et al., 2020; Piercy et al., 2018).

### 3.2 Arterial waveform analysis and hot flashes

**FIGURE 1:**
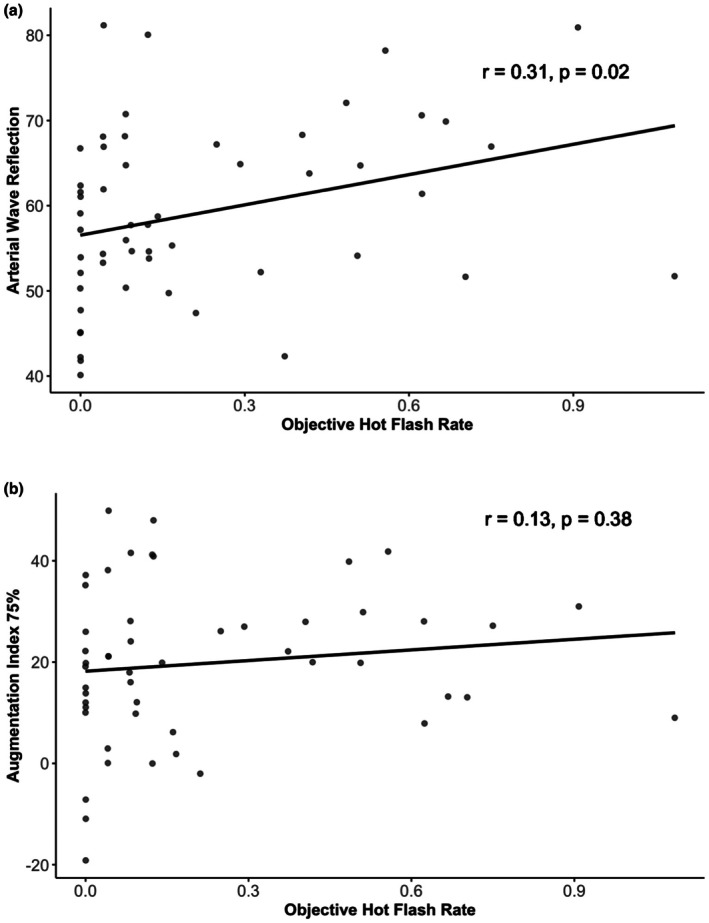
Relationship between arterial stiffness and objective hot flash rate. (a) Arterial Wave Reflection, (b) Augmentation Index 75.

Higher WR but not AIx75 was significantly correlated with higher objective HF rate [WR: *r* (51)=0.31, *p* = 0.02 and AIx75: *r* (49)=0.13, *p* = 0.38] (Figure 1). When WR was further broken down into P_f_ and P_b_, only P_b_ but not P_f_ was significantly correlated with HF [*P*_b_: *r* (51)=0.32, *p* = 0.02 and *P*_f_: *r* (51)=0.15, *p* = 0.28]. Correlation results evaluated with nonparametric Spearman rank correlation tests yielded similar results [WR: *ρ* = 0.34, *p* = 0.01, *P*_b_: *ρ* = 0.39, *p* = 0.004, *P*_f_: *ρ* = 0.16, *p* = 0.26, and AIx75: *ρ* = 0.20, *p* = 0.16]. No significant correlation was observed between HF rate and PWV (*r* (46) = 0.08, *p* = 0.58).

Multiple regression results for predicting arterial waveform variables using objective HF rate are reported in Table 2. Objective HF rate significantly predicted WR (*ꞵ*[SE] = 10.05[4.33], *p* = 0.03; model 3); however, that was not the case for AIx75 (*ꞵ*[SE] = 7.39[6.59], *p* = 0.3; model 1) or AIx (*ꞵ*[SE] = 7.67[5.88], *p* = 0.2; model 2). BMI was significantly associated with all of the arterial waveform variables in all models.

**TABLE 2:** Regression models predicting augmentation indexes and arterial wave reflection variables using objective hot flash rate.

Variables	AIx75	AIx	WR
	Model 1 (***)	Model 2 (***)	Model 3 (***)
HF	7.39	7.67	10.1**
Age	0.54	0.34	0.21
BMI	1.71***	1.46***	0.92***
SBP	0.01	0.16	0.19
*R* ^2^	0.39	0.41	0.42
Adj *R* ^2^	0.34	0.36	0.37

** *p* ≤ 0.05.

*** *p* ≤ 0.001.

### 3.3 Arterial waveform analysis and physical activity

**FIGURE 2:**
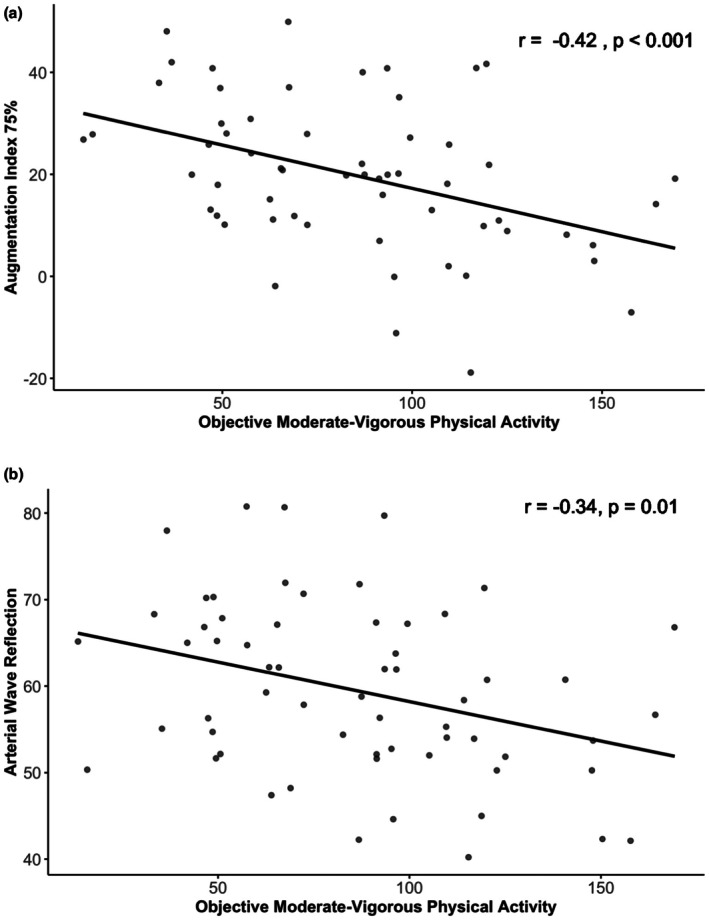
Relationship between arterial stiffness and objective moderate‐vigorous physical activity. (a) Augmentation Index 75, (b) Arterial Wave Reflection.

**FIGURE 3:**
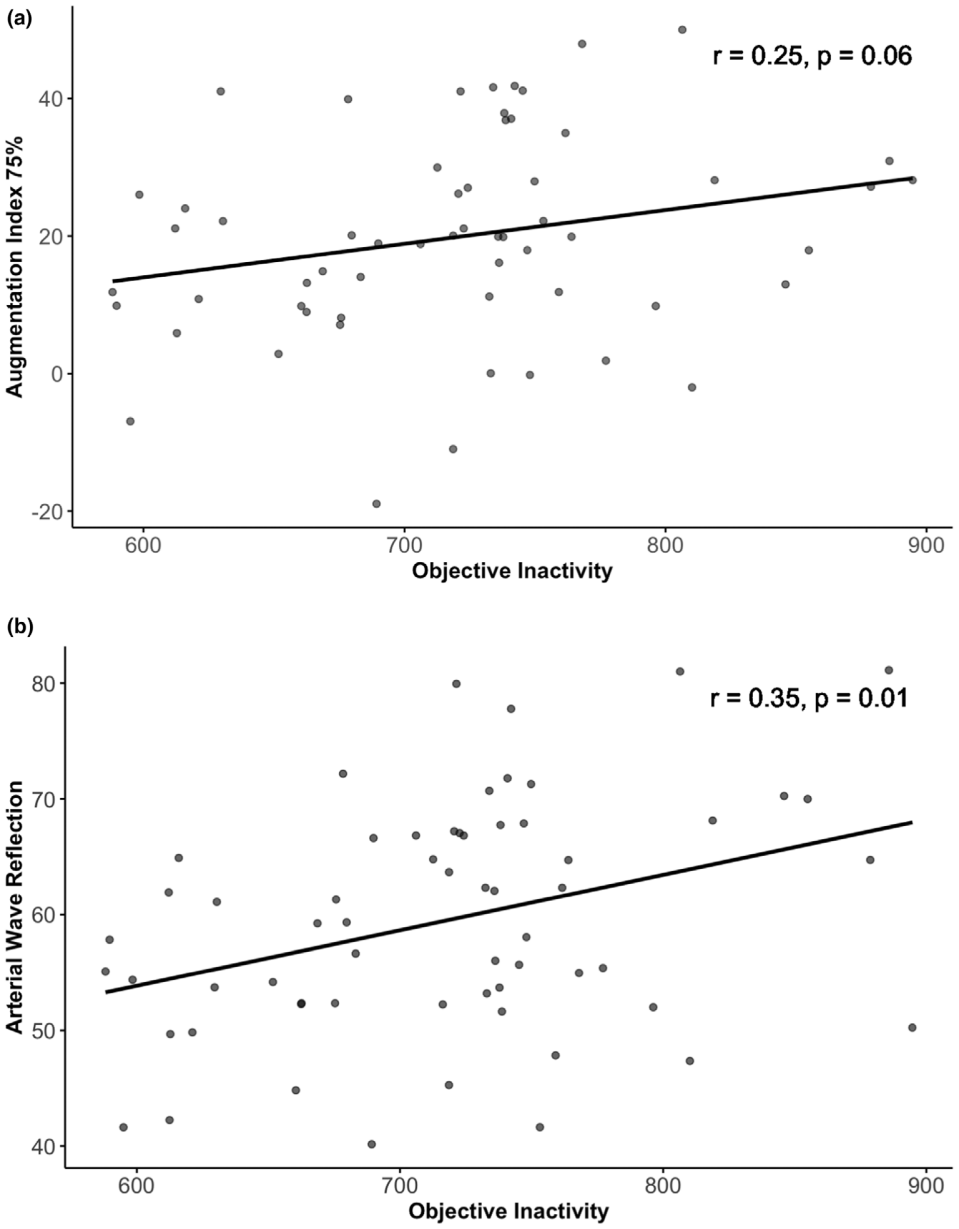
Relationship between arterial stiffness and objective inactivity. (a) Augmentation Index 75, (b) Arterial Wave Reflection.

There was a significant inverse relationship between MVPA and both AIx75 (*r* (57) = − 0.42, *p* = 0.0009) and WR (*r* (59) = − 0.34, *p* = 0.008) (Figure 2). Conversely, higher WR was associated with more inactivity minutes [WR: *r* (59) = 0.35, *p* = 0.005] (Figure 3).

Multiple regression models predicting AIx75 using PA variables are shown in Table 3a. MPA (*ꞵ*[SE] = −0.12[0.05], *p* = 0.03; model 3), VPA (*ꞵ*[SE] = −0.34[0.11], *p* = 0.004; model 4), and MVPA (*ꞵ*[SE] = −0.13[0.04], *p* = 0.004; model 5) were significant predictors of AIx75, controlling for age, BMI, and BP. The regression results for WR with different PA levels are reported in Table 3b. Among the five objectively measured PA level variables, VPA (*ꞵ*[SE] = −0.17[0.08], *p* = 0.03, model 4) and MVPA (*ꞵ*[SE] = −0.06[0.03], *p* = 0.05, model 5) had significant associations with WR, controlling for age, BMI, and BP while inactivity demonstrated a trend (*ꞵ*[SE] = 0.03[0.02], *p* = 0.08, model 1). BMI was significantly associated with both AIx75 and WR as well as SBP with WR in all models.

**TABLE 3a:** Regression models predicting augmentation index 75.

Variables	AIx75				
	Model 1 (***)	Model 2 (***)	Model 3 (***)	Model 4 (***)	Model 5 (***)
Inactivity	0.03				
LPA		−0.01			
MPA			−0.12**		
VPA				−0.34**	
MVPA					−0.13**
Age	0.39	0.26	0.36	−0.02	0.29
BMI	1.56***	1.61***	1.51***	1.35***	1.40***
SBP	0.07	0.18	0.11	0.17	0.13
*R* ^2^	0.36	0.35	0.40	0.44	0.44
Adj *R* ^2^	0.31	0.30	0.36	0.40	0.40

Abbreviation: ns, not significant.

** *p* ≤ 0.05.

*** *p* ≤ 0.001.

**TABLE 3b:** Regression models predicting arterial wave reflection.

Variables	WR				
	Model 1 (***)	Model 2 (***)	Model 3 (***)	Model 4 (***)	Model 5 (***)
Inactivity	0.03*				
LPA		−0.004			
MPA			−0.05		
VPA				−0.17**	
MVPA					−0.06**
Age	0.12	−0.07	0.001	−0.13	−0.006
BMI	0.79***	0.86***	0.81***	0.72***	0.75***
SBP	0.21**	0.25**	0.25**	0.26**	0.25**
*R* ^2^	0.39	0.35	0.38	0.40	0.40
Adj *R* ^2^	0.34	0.31	0.33	0.36	0.35

Abbreviation: ns, not significant.

* *p* < 0.1.

** *p* ≤ 0.05.

*** *p* ≤ 0.001.

### 3.4 Interaction of physical activity and hot flash on arterial wave reflection

Of the five different PA engagement levels, only moderate‐intensity physical activity influenced the association between hot flash and arterial wave reflection (Table 4). The interaction was such that MPA levels significantly moderated the relationship between HF and WR, and this interaction was observed independently of covariate inclusion (Table 4, model 2 and model 4). The negative coefficient indicated that at higher MPA levels, the association between HF and WR was weakened. There was no influence of MVPA nor VPA engagement on the relationship between HF and WR, suggesting that MPA may be the most beneficial PA intensity to reduce the negative HF and WR relationship for perimenopausal females.

**TABLE 4:** Regression models including interaction predicting arterial wave reflection.

Variables	WR			
	Model 1 (**)	Model 2 (**)	Model 3 (***)	Model 4 (***)
HF	10.80**	41.46**	9.44**	30.36**
MPA	−0.09**	−0.01	−0.07*	−0.01
HF × MPA		−0.37**		−0.25*
Age			0.31	0.22
BMI			0.85***	0.80***
SBP			0.18	0.18
*R* ^2^	0.16	0.24	0.46	0.49
Adj *R* ^2^	0.13	0.19	0.40	0.42

* *p* < 0.1.

** *p* ≤ 0.05.

*** *p* ≤ 0.001.

**FIGURE 4:**
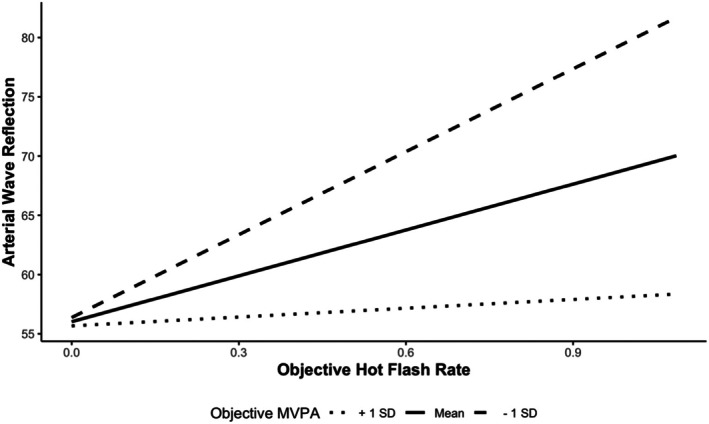
Relationship between arterial wave reflection and objective hot flash rate differs at lower and higher objective moderate physical activity.

Further simple slopes analysis of the interaction effect between HF and MPA showed that the correlation between HF and WR at low MPA (−1SD from mean MPA) was significantly positive (*ꞵ*[SE] = 23.33[7.52], *p* = 0.003), whereas this effect was absent at high MPA (+1SD from mean MPA, *ꞵ*[SE] = 2.48[6.14], *p* = 0.69, Table 5). We also found that the correlation between HF and WR at sample mean MPA value was significant (*ꞵ*[SE] = 12.90[4.88], *p* = 0.01), although this relationship was weaker compared to lower levels of MPA (Figure 4).

**TABLE 5:** Simple slopes analysis predicting arterial wave reflection.

Dependent variable	Moderator (±1 SD)	df	HF β	SE	*t*	*p*	95% CI
WR	Low MPA (49.72)	49	23.33	7.52	3.10	0.003	[8.21, 38.45]
	Mean MPA (78.32)	49	12.90	4.88	2.65	0.011	[3.10, 22.71]
	High MPA (106.92)	49	2.48	6.14	0.40	0.689	[6.14, −9.87]

*Note*: Low MPA = MPA—mean (MPA) + 1 SD; Mean MPA = MPA—mean (MPA); High MPA = MPA—mean (MPA)—1 SD.

## 4 DISCUSSION

Accumulating evidence suggests HF are associated with CVD risk. In a healthy perimenopausal population with no evidence of CVD risk, results of the current study showed that higher WR was correlated with higher objectively measured HF and HF was a significant predictor of WR in regression models controlling for age, BMI, and systolic blood pressure. Physical activity was also related to WR as higher amounts of habitual moderate and vigorous physical activity were related to lower WR, and greater physical inactivity time was correlated with higher WR. Finally, to the best of our knowledge, we report for the first time that higher levels of MPA moderated the significant relationship between HF and WR in healthy perimenopausal females, whereas the negative association between HF and WR was significant at lower levels but not at higher levels of moderate PA. Overall, these results support recommendations for engagement in moderate and vigorous physical activity and reducing inactive time for perimenopausal females to mitigate potential changes in CVD risk associated with hot flash symptoms.

Hot flashes have been associated with a variety of clinical and subclinical CVD risk factors (Carson & Thurston, 2023; Thurston, 2024) with early effects on blood vessel function showing up in younger midlife (Thurston et al., 2018), and perimenopausal females (Thurston et al., 2017; Witkowski et al., 2025). With the data presented herein, we add to this body of evidence, showing that in healthy perimenopausal females with normal CVD risk factors, WR was associated with objectively measured HF. Higher backward pressure waves increase WR and originate from bifurcations and differences in impedance in the arterial tree (Chirinos & Segers, 2010; Nichols & Singh, 2002). Arterial stiffness can also influence reflected pressure wave amplitude (Nichols, 2005). Higher backward wave reflection and WR are considered to be detrimental to the cardiovascular system as the reflected pressure waves create greater cardiac load (Weber et al., 2012). Importantly, we did not find any association between HF and PWV, a direct measurement of arterial stiffness, which may indicate that impedance mismatch or vessel diameter underlies the association with HF in this healthy perimenopausal population. Others have reported associations between arterial stiffness via PWV and subjectively‐assessed HF (Yang et al., 2017) however this study included individuals with CVD risk factors, such as hypertension or diabetes, and/or reproductive abnormalities such as uterine fibroids and endometriosis. Mechanisms connecting HF to CVD risk factors are uncertain but may include autonomic cardiovascular control, HPA axis activity, inflammation, and coagulation factors (Carson & Thurston, 2023; Thurston, 2024). Interestingly, as found in the current study, hormone levels do not appear to explain the relationships (Gast et al., 2010, 2011; Thurston et al., 2016; Witkowski et al., 2025).

Existing studies in middle‐aged and younger populations report protective effects of PA on CVD. Dempsey et al. (Dempsey et al., 2022) using data from UK Biobank middle‐aged adults (40–69 years old) without prevalent CVD, showed that higher PA energy expenditure and higher %MVPA (i.e., the fraction of PA energy expenditure from MVPA) were associated with lower rates of incident CVD. The risk was lower in more intense PA, supporting the important role of PA in reducing future CVD risk. In Coronary Artery Risk Development in Young Adults (CARDIA) study, Full et al. reported that replacing 24 min of physical inactivity time with MVPA reduced the odds of CVD risk and functional burden by 15% in both males and females (OR: 0.85; 95% CI, 0.75–0.96) while reallocating 24 min of LPA to MVPA decreased the risk by 14% (OR: 0.86; CI: 0.75, 0.99) (Weber et al., 2012). A meta‐analysis of 42 randomized controlled trials showed that aerobic exercise interventions significantly improved PWV and AIx, where the effect was enhanced in participants with higher exercise intensity and greater arterial stiffness at baseline (Ashor et al., 2014). The findings herein suggest that regular moderate to vigorous exercise is associated with reduced WR and AIx75 in perimenopausal females.

The relations between PA and arterial wave reflection may be explained by the functional anatomy of blood vasculature. The aorta is the central artery that regulates the pulsatile blood flow from the heart into the steady flow needed to supply peripheral organs. Improper matching between aortic diameter and flow can increase forward arterial pressure wave amplitude and pulse pressure, promoting an earlier arrival of the reflected waves (Chirinos et al., 2019; Kaess et al., 2012). Prior research has highlighted that exercise can induce structural remodeling of the arteries, contributing to proper artery diameter, pressure, and functioning and reduction in artery wall thickness (Thijssen et al., 2011). Acute exercise has also been shown to reduce WR (Gast et al., 2010; Thurston et al., 2016) and AIx (Kingsley & Figueroa, 2012), and has been attributed to reductions in vascular resistance (Stock et al., 2021). Participants in the current study were asked to refrain from exercise 12 h prior to testing and exercise engagement was stable for at least the prior 2 years. Therefore, it is feasible that lower resting WR and AIx in more physically active participants result from regular engagement in moderate to vigorous activity. Regular engagement in PA may promote structural changes and reduce mismatch via lowering vascular resistance.

In this study, higher physical inactivity time was associated with increased WR independent of other PA levels or CVD risk factors. Studies on physical inactivity in females have focused on older postmenopausal females who have ceased menstrual periods for at least 1 year. Chomistek et al. (Chomistek et al., 2013), reported that in postmenopausal females without a history of CVD, lower leisure‐time PA was strongly associated with increased CVD risk after adjusting for sitting time. Furthermore, physically inactive participants reporting 10 h/day physical inactivity time had 63% greater CVD risk compared to those with 5 h/day physical inactivity time. Bellettiere et al. (Bellettiere et al., 2019), found that females with >11 h/day physical inactivity time had higher risk for CVD (HR: 1.62; 95% CI, 1.21–2.17; *p* < 0.001) compared to those with <9 h/day physical inactivity time in the older postmenopausal population. Females having both high physical inactivity time and long bout durations of physical inactivity time had significantly higher risk for CVD (HR: 1.34; 95% CI, 1.08–1.65) compared to those with low physical inactivity time and short bout durations. Thus, higher physical inactivity time may be a risk factor for CVD regardless of other strenuous or moderate PA levels and should be limited in perimenopausal people.

Current PA recommendations suggest 150–300 mins of MPA, 75–150 mins of VPA, or an equivalent combination of two intensities with >2 days of strength/resistance training per week (Baber & Panay, 2016; Bull et al., 2020; Piercy et al., 2018). Furthermore, current PA guidelines for Americans emphasize that “moving more and sitting less will benefit nearly everyone” (Piercy et al., 2018). Our study population generally maintained a PA level much higher than recommended (86 mins/day ±37). According to our simple slope analysis, there is a positive association between worsening WR and higher HF rate at lower MPA levels (50 mins/day). However, at higher MPA levels (107 mins/day), this association is eliminated. Thus, while general PA can improve health outcomes, our findings emphasize that amounts of moderate intensity PA levels higher than current recommendations may play the most important role in minimizing WR changes associated with HF in perimenopausal females over any other PA intensity.

Aging and other CVD risk factors, such as central adiposity, hyperlipidemia, hypertension, and impaired glucose tolerance, escalate arterial dysfunction by accumulating collagen deposition and reducing vascular elasticity. In this study, we found that BMI was significantly associated with arterial waveform variables, that is, AIx75 and WR, in regression models. Such a relationship has been reported between BMI and arterial stiffness previously. Similar to our study, most studies highlight the role of obesity in accelerating arterial stiffness progression (Piko et al., 2023; Tang et al., 2020). Hormonal changes are inevitable during the perimenopause transition. Females experience decreasing circulating estrogen (e.g., E2) and increasing gonadotropic hormones (e.g., FSH) and these changes have been associated with changes in energy expenditure and energy intake leading to a positive energy balance and weight gain (Marlatt et al., 2022). When we tested for whether hormone changes were associated with WR, AIx or AIx75, no significant correlation was found. Similar results were reported by Samargandy et al., where they found that these hormones did not explain any difference in arterial stiffness for females undergoing the menopause transition (Samargandy et al., 2020).

### 4.1 Significance and limitations

Several strengths of this study are worth mentioning. First, this research focuses on perimenopause, the critical period when CVD risk appears to increase dramatically. Second, we employed objective measures of PA and HF, increasing the reliability of our data acquisition. Despite these strengths, there are some limitations. The observational cross‐sectional study design only allows for association testing and may limit the understanding of causal relationships. Second, this study is limited by a small sample size of healthy participants mostly with European ancestry. Participants were asked to refrain from exercise for only 12 h prior to testing. The post‐exercise hypotensive response can persist for up to 22 h and may have influenced blood pressure assessment in the study. Further, on average, they are more physically active than what has been reported for this population. Therefore, future longitudinal studies should confirm these relationships in a more diverse population.

### 4.2 Conclusions

Engaging in more moderate physical activity and reducing physical inactivity time may play an important role in reducing the effect of hot flash symptoms on CVD risk in perimenopause.

## AUTHOR CONTRIBUTIONS

**Tint Tha Ra Wun:** Data curation; methodology; visualization. **Jo Sophia Brunzelle:** Data curation; formal analysis. **Lorna Murphy:** Data curation; project administration. **Randi L. Garcia:** Formal analysis; supervision. **Lynnette Leidy Sievert:** Methodology; supervision. **Sarah Witkowski:** Conceptualization; data curation; formal analysis; funding acquisition; investigation; methodology; project administration; resources; software; supervision; validation.

## FUNDING INFORMATION

This research was supported by NIH (NHLBI) 1R15HL145650‐01A1 (Witkowski) and Smith College McKinley Fellowship (Tha Ra Wun).

## CONFLICTS OF INTEREST STATEMENT

Authors have no conflicts of interest.

## ETHICS STATEMENT

This study complied with the ethical principles established by the Institutional Review Board at Smith College (Approval Number: 18‐108). All participants reviewed and were required to provide written informed consent before any study procedures.

## DISCLAIMERS

None.

## Data Availability

The data that support the findings of this study are available from the corresponding author upon reasonable request.
